# Role of lncRNA MIAT/miR-361-3p/CCAR2 in prostate cancer cells

**DOI:** 10.1515/med-2021-0380

**Published:** 2022-09-28

**Authors:** Tao Feng, Chunyu Song, Zhiyong Wu, Ke Zhao, Shenglan Ye

**Affiliations:** Department of Urology, The Central Hospital of Wuhan, Tongji Medical College, Huazhong University of Science and Technology, Wuhan, 430014, China; Department of Endocrine, The Central Hospital of Wuhan, Tongji Medical College, Huazhong University of Science and Technology, Wuhan, 430014, China; Department of Nail and Breast Surgery, The Central Hospital of Wuhan, Tongji Medical College, Huazhong University of Science and Technology, Wuhan, 430014, China; Department of Thoracic Surgery, The Central Hospital of Wuhan, Tongji Medical College, Huazhong University of Science and Technology, No. 26 Shengli Street, Wuhan, 430014, China; Department of Respiratory, The Central Hospital of Wuhan, Tongji Medical College, Huazhong University of Science and Technology, No. 26 Shengli Street, Wuhan, 430014, China

**Keywords:** prostate cancer, lncRNA MIAT, miR-361-3p, CCAR2

## Abstract

The study was aimed to investigate the role and mechanism of long non-coding RNAs (lncRNA) myocardial infarction-associated transcript (MIAT) in prostate cancer. The relationships between lncRNA MIAT and miR-361-3p, miR-361-3p and cell cycle and apoptosis regulator 2 (CCAR2) were predicted by StarBase and TargetScan, and verified by dual-luciferase reporter assay and RNA pull-down assay. Quantitative real-time PCR assay was performed to detect the mRNA expression of lncRNA MIAT, miR-361-3p, CCAR2, Bax, and Bcl-2 in the prostate cancer tissues or cells. The protein levels of CCAR2, Bax, and Bcl-2 were detected by Western blot analysis. The cell viability and apoptosis were detected by MTT assay and Flow cytometry analysis, respectively. lncRNA MIAT was upregulated, while miR-361 was downregulated in the prostate cancer tissues and Du145 cells. lncRNA MIAT negatively regulated miR-361-3p expression in Du145 cells. Downregulating lncRNA MIAT decreased the cell viability, induced the cell apoptosis, increased Bax expression, and decreased Bcl-2 expression in Du145 cells, while the effects were reversed by downregulating miR-361-3p or CCAR2 upregulation. Moreover, CCAR2 upregulation reversed the effects of miR-361-3p upregulation on Du145 cell viability and apoptosis. In conclusion, lncRNA MIAT participated in prostate cancer by regulating cell proliferation and apoptosis via miR-361-3p/CCAR2 axis.

## 1 Introduction

Prostate cancer, a malignant tumor caused by the proliferation of prostate epithelial cells, is one of the most common malignant tumors of the male reproductive system, and its incidence increases with age [1,2]. According to cell type and origin classification, prostate cancer can be divided into prostate adenocarcinoma, transitional cell epithelial carcinoma such as ductal adenocarcinoma, urothelial carcinoma, squamous cell carcinoma, and adenosquamous carcinoma [3,4]. Among them, prostate adenocarcinoma accounts for more than 95%, which is currently the most common prostate cancer [5]. Most early prostate cancer has no obvious symptoms. As the tumor grows, prostate cancer can manifest as symptoms of lower urinary tract obstruction, such as frequent urination, urgency, slow urinary flow, labored urination, and even urinary retention or incontinence. When tumor metastases to bone, it can cause symptoms such as bone pain, spinal cord compression, and pathological fractures [6,7]. At present, the pathogenic factors of prostate cancer are not completely clear, but studies have shown that it may be related to age, race, genetics, environment, food, obesity, and sex hormones [8,9]. With the rapid increase in the incidence of prostate cancer in China, it is of great significance to optimize the prevention, diagnosis, and treatment of prostate cancer.

Long non-coding RNAs (lncRNAs) are a class of RNAs with length of more than 200 nt [10]. They regulate the expression of genes at various levels in the form of RNAs, including epigenetic regulation, transcription regulation, and post-transcriptional regulation, but do not encode proteins [11,12]. It is known that lncRNAs are widely involved in various biological processes, and their abnormal expression is closely related to many diseases. Recently, lncRNAs have been reported as the important regulators in prostate cancer [13,14]. Myocardial infarction-associated transcript (MIAT), one of major lncRNAs associated with cancer process, has huge application prospects in clinical applications and is expected to become a new tumor biomarker and therapeutic target [15]. lncRNA MIAT has been found to be in abnormal expression in different tumors, including gastric cancer, cervical cancer, breast cancer, and lung cancer [16,17,18,19]. However, there are few reports on the role of lncRNA MIAT in prostate cancer.

Previous studies have found that the expression of miR-361-3p was significantly increased and cell cycle and apoptosis regulator 2 (CCAR2) was decreased in prostate cancer [20,21], and miR-361-3p/CCAR2 play critical roles in the regulation of the proliferation and apoptosis of prostate cancer cells. What was exciting is that through bioinformatics analysis, we found that lncRNA MIAT may sponge miR-361-3p, and CCAR2 may be the direct target gene of miR-361-3p. Therefore, we assumed that lncRNA MIAT may affect the proliferation and apoptosis of prostate cancer cells by regulating miR-361-3p/CCAR2, and play a certain role in prostate cancer.

In the study, we investigated the expression of lncRNA MIAT in prostate cancer, and explored whether it can affect the proliferation and apoptosis of prostate cancer cells by regulating the miR-361-3p/CCAR2 axis, so as to provide new targets for the treatment of prostate cancer.

## 2 Materials and methods

### 2.1 Tissue acquisition

30 cases of prostate cancer tissues and 30 cases of adjacent nontumor tissues were collected from male patients who underwent tumor resection at the Central Hospital of Wuhan. These patients were diagnosed with prostate cancer and did not receive radiotherapy, chemotherapy, or immunotherapy before surgery. The clinicopathologic characteristics of prostate cancer patients are presented in Table 1. The study was approved by the ethics committee of the Central Hospital of Wuhan, and informed patient consent was obtained before tissue collection.

**Table 1:** Clinicopathologic characteristics of prostate cancer patients

Parameter	Cases (*n* = 30)
Age (years)	
<60	12
≥60	18
T stage	
T1–T2	22
T3–T4	8
Lymph node metastasis	
No	17
Yes	13
Distance metastasis	
No	16
Yes	14

### 2.2 Cell culture

Human prostate cancer cell line Du145 (ATCC^®^ HTB-81; ATCC, USA) and normal human prostate epithelial cells RWPE-2 (cat. no. ATCC^®^ CRL-11610™; ATCC, USA) were obtained for this research. Du145 cells were cultured in Eagle’s minimum essential medium (cat. no. 30-2003™; ATCC, USA) containing 10% fetal bovine serum (cat. no. 10091155; Gibco, USA), and RWPE-2 cells were cultured in keratinocyte serum free medium (cat. no. 17005-042; Gibco, USA) with 0.05 mg/mL bovine pituitary extract and 5 ng/mL human recombinant epidermal growth factor. All the cells were incubated in 37°C and 5% CO_2_.

### 2.3 miRNA target analysis and dual-luciferase reporter assay

Bioinformatics software StarBase and TargetScan were used to identify the relationships between the lncRNA MIAT and miR-361-3p, CCAR2 and miR-361-3p, respectively. The relationships were verified by dual-luciferase reporter assay. For example, we obtained the 3′-UTR product of lncRNA MIAT, including the target sequence of miR-361-3p, and fused the 3′-UTR product with the pmirGLO vector (cat. no. E1330; Promega, USA) to construct the reporter vector lncRNA MIAT wild Type (lncRNA MIAT-WT). Then, we formed the vector lncRNA MIAT mutant (lncRNA MIAT-MUT). A QuikChange Site-Directed Mutagenesis Kit (Stratagene; Agilent Technologies, Inc.) was applied according to the manufacturer’s instructions to point-mutate the miR-361-3p-binding domain in the 3′-UTR of lncRNA MIAT. 293T cells cultured over 24 h were co-transfected with MIAT-WT or MIAT-MUT and miR-361-3p mimic or mimic control through Lipofectamine 2000 reagent (cat. no. 11668030; Invitrogen, USA) for 48 h. The luciferase activity was analyzed by using a dual-luciferase reporter gene analysis system (cat. no. E1910; Promega, USA).

### 2.4 RNA pull-down assay

The pull-down assay was applied as previously described. Briefly, the biotinylated miR-361-3p probe and oligo probe (GenePharma, China) were incubated with M-280 Streptavidin magnetic beads (cat. no. 60210; Invitrogen, USA) for 2 h at room temperature to generate probe-coated beads. Then, Du145 cells (10^7^ cells) were harvested, lysed, sonicated, and incubated with probe-coated beads at 4°C overnight. After washing, the RNA complexes bound to the beads were eluted and extracted with RNA isolation kit (cat. no. AM1920; Invitrogen, USA) and analyzed by quantitative real-time PCR (qRT-PCR) assay.

### 2.5 Cell transfection assay

Du145 cells were seeded into 6-well plates and cultured overnight, and then transfected with control-siRNA, lncRNA MIAT-siRNA, inhibitor control, miR-361-3p inhibitor, control-plasmid, CCAR2-plasmid, mimic control, or miR-361-3p mimic by using Lipofectamine^™^ 2000 Transfection Reagent (Invitrogen, USA). Then, the cells were collected to detect the transfection efficiency by qRT-PCR assay.

### 2.6 RNA extraction and qRT-PCR

Total RNA was extracted from the tissues and cells by using TRIzol reagent (cat. no. AM1920; Invitrogen, USA), and was reverse transcribed into cDNA by using cDNA synthesis kit (cat. no. K1612; Thermo Fisher, USA). cDNA was used for qRT-PCR analysis by using ChamQ^TM^ Universal SYBR^®^ qPCR Master Mix (cat. no. Q711-02/03; Vazyme, China). The primers were synthesized by Sangon (Shanghai, China). The relative mRNA expression levels of MIAT, miR-361-3p, Bax, Bcl-2, and CCAR2 were calculated by 2^−ΔΔCt^ analysis.

### 2.7 MTT assay

MTT assay was performed to measure cell viability. Cells were spread into a 96-well plate overnight. 10 μL of MTT solution (cat. no. C0009M; Beyotime, China) was added to each well and incubated with cells for 4 h. 100 μL of DMSO was added to each well to dissolve the formazan product after the medium was removed. The absorbance was recorded at 570 nm with a microplate reader (Bio-Rad, CA, USA) in 10 min.

### 2.8 Flow cytometry (FCM) analysis

FCM analysis was used to measure cell apoptosis. Cells were collected and re-suspended in 1× buffer. 100 µL of cell suspension was incubated with 5 µL Annexin V-FITC and 5 μL of PI (cat. no. C1062M; Beyotime Institute of Biotechnology, Shanghai, China). The stained cells were analyzed by FACSCalibur flow cytometer (BD, USA), and the data were analyzed by FlowJo software.

### 2.9 Western blot analysis

Du145 cells were lysed with RIPA buffer (cat. no. P0013C; Beyotime, Nanjing, China), and centrifuged to obtain total protein. The concentration of the protein was determined by BCA protein kit (Bio-Rad, USA). The protein was separated by SDS-PAGE, and then transferred to PVDF membrane. The membrane was incubated with Bcl-2 (1:1,000; cat. no. 4223; Cell Signaling Technology, Inc.), Bax (1:1,000; cat. no. 5023; Cell Signaling Technology, Inc.), or GAPDH (1:1,000; cat. no. 5174; Cell Signaling Technology, Inc.) antibody at 4°C overnight after blocking with 5% non-fat milk PBST solution for 1 h. After that, the membrane was incubated with the secondary antibody (cat. no. ab97080; Abcam, USA) for 1 h at room temperature. The protein bands were visualized by ECL luminescent substrate (Cytiva, Amersham ImageQuant 800UV, USA), and the results were analyzed by ImageJ software.

### 2.10 Statistical analysis

Statistical analysis was performed using SPSS 20.0 (IBM Corp.). All data were showed as the mean value ± standard deviation (SD). The statistical analysis between each group was analyzed by student’s *t* test or one-way analysis of variance followed by Tukey’s *post hoc* test. A *p* value < 0.05 (*p* < 0.05) was considered significant.

## 3 Results

### 3.1 miR-361-3p targeted lncRNA MIAT

**Figure 1:**
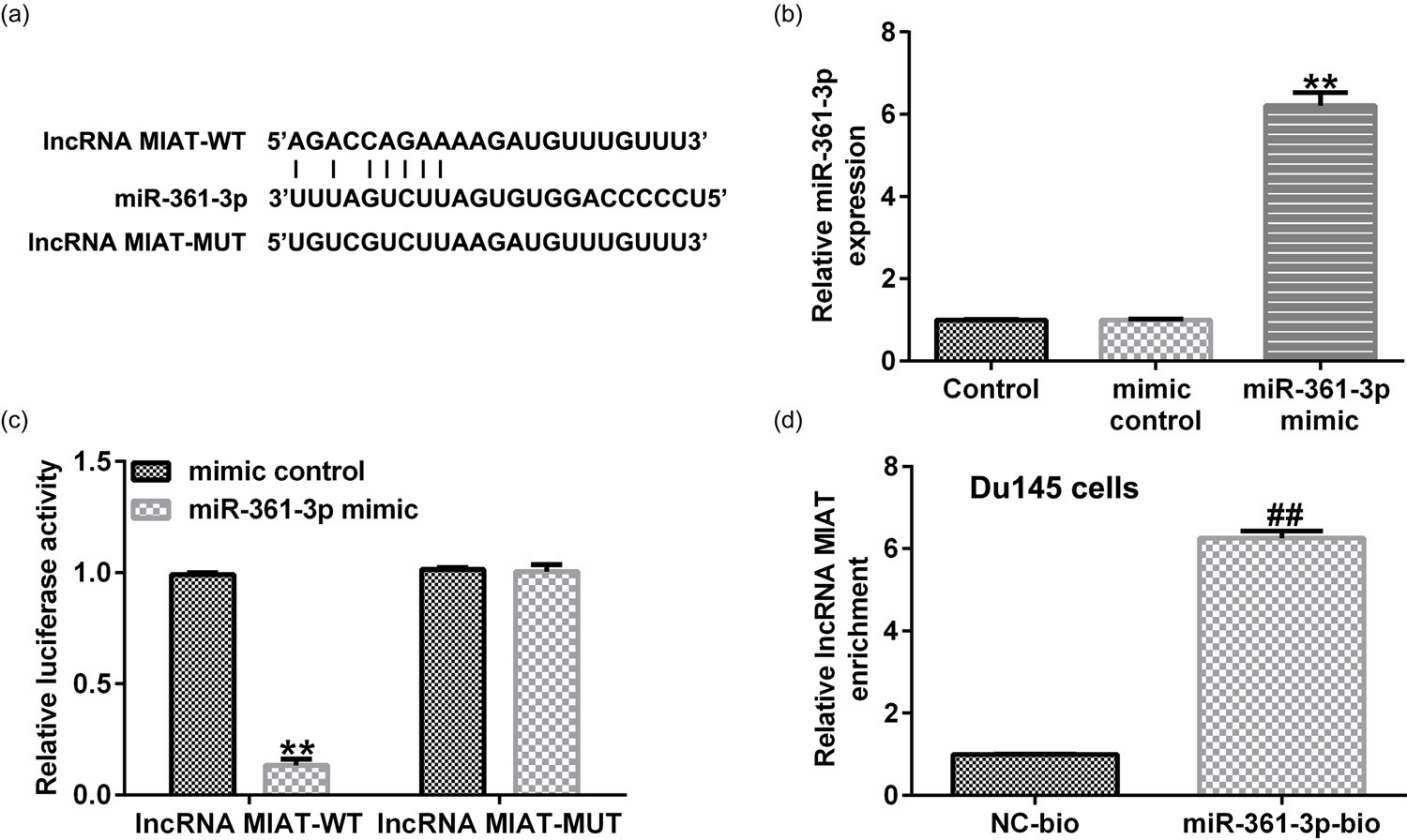
miR-361-3p directly targeted lncRNA MIAT. (a) Starbase analysis predicted the binding site between lncRNA MIAT and miR-361-3p. (b) The level of miR-361-3p in 293 T cells transfected with mimic control or miR-361-3p mimic was determined using qRT-PCR. (c) Dual-luciferase reporter assay verified the relationship between lncRNA MIAT and miR-361-3p. (d) RNA pull-down assay verified the binding site between lncRNA MIAT and miR-361-3p. All experiments were repeated three times, and the data are shown as the mean value ± SD. **p < 0.01 vs normal adjacent tissue; ##*p* < 0.01 vs RWPE-2.

The result of Starbase analysis showed that there was a binding site between lncRNA MIAT and miR-361-3p (Figure 1a). We first confirmed that compared with the mimic control group, miR-361-3p mimic significantly enhanced miR-361-3p expression in 293 T cells (Figure 1b). Besides, the binding sites between lncRNA MIAT and miR-361-3p were confirmed by dual-luciferase reporter assay (Figure 1c) and RNA pull-down analysis (Figure 1d).

### 3.2 Expression of lncRNA MIAT and miR-361-3p in prostate cancer tissues and cells

**Figure 2:**
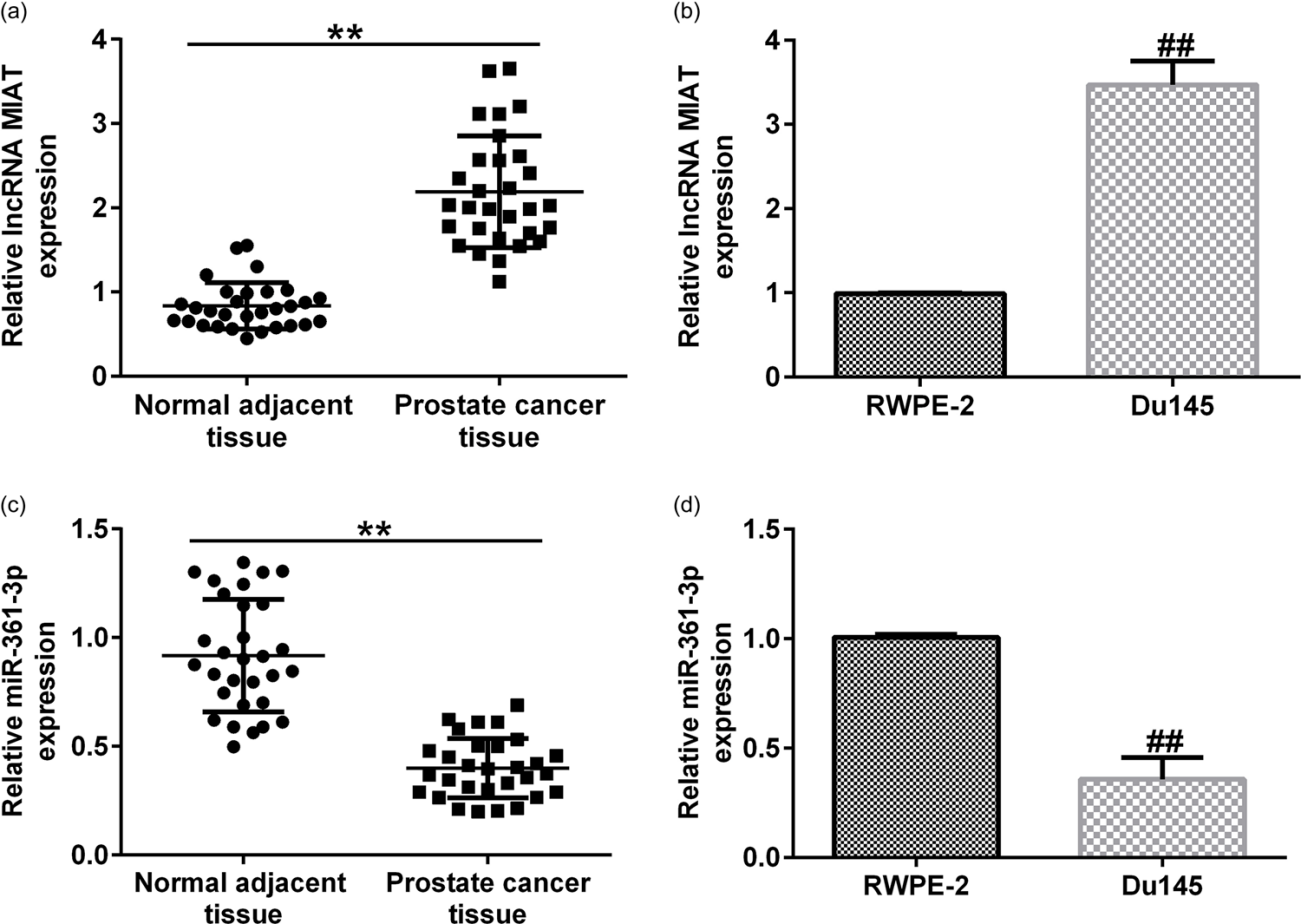
Expression of lncRNA MIAT and miR-361-3p in prostate cancer tissues and cells. (a and b) qRT-PCR analysis was performed to measure the mRNA expression of lncRNA MIAT in prostate cancer tissues and cells. (c and d) qRT-PCR analysis was performed to measure the mRNA expression of miR-361-3p in prostate cancer tissues and cells. All experiments were repeated three times, and the data are shown as the mean value ± SD. **p < 0.01 vs mimic control; ##*p* < 0.01 vs NC-bio.

As the results showed that, compared with the adjacent tissues, the expression of lncRNA MIAT in prostate cancer tissues was significantly upregulated (Figure 2a). Similarly, the expression of lncRNA MIAT in the prostate cancer cell line Du145 cells was significantly increased compared with normal human prostate epithelial cells RWPE-2 cells (Figure 2b). On the contrary, miR-361-3p expression was downregulated in prostate cancer tissues and Du145 cells compared with the control (Figure 2c and d).

### 3.3 lncRNA MIAT negatively regulated miR-361-3p expression in Du145 cells

**Figure 3:**
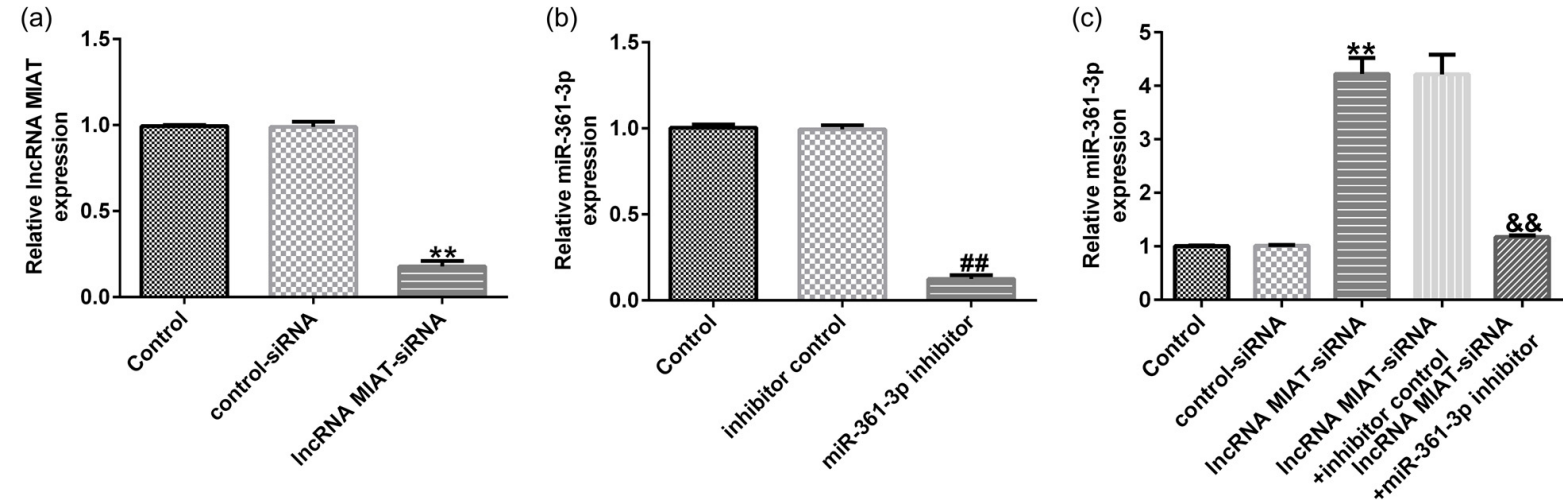
Effects of lncRNA MIAT-siRNA on the expression of miR-361-3p in Du145 cells. (a) qRT-PCR analysis was used to detect the mRNA expression of lncRNA MIAT in Du145 cells transfected with control-siRNA or lncRNA MIAT-siRNA. (b) qRT-PCR analysis was used to detect the expression of miR-361-3p in Du145 cells transfected with inhibitor control or miR-361-3p inhibitor. (c) qRT-PCR analysis was used to detect the expression of miR-361-3p in Du145 cells transfected with control-siRNA, lncRNA MIAT-siRNA, lncRNA MIAT-siRNA + inhibitor control, or lncRNA MIAT-siRNA + miR-361-3p inhibitor. All experiments were repeated three times, and the data are shown as the mean value ± SD. **p < 0.01 vs control-siRNA; ##*p* < 0.01 vs inhibitor control; &&*p < 0.01 vs lncRNA MIAT-siRNA + inhibitor control.

We detected the expression of lncRNA MIAT and miR-361-3p in Du145 cells after the cell transfection. The results showed that lncRNA MIAT-siRNA and miR-361-3p inhibitor markedly reduced the expression of lncRNA MIAT and miR-361-3p, respectively, in Du145 cells (Figure 3a and b). In addition, in comparison with the control-siRNA group, lncRNA MIAT-siRNA significantly increased the expression of miR-361-3p in Du145 cells, and the increase was significantly reversed by miR-361-3p inhibitor (Figure 3c).

### 3.4 lncRNA MIAT-siRNA affected the growth of Du145 cells by regulating miR-361-3p

**Figure 4:**
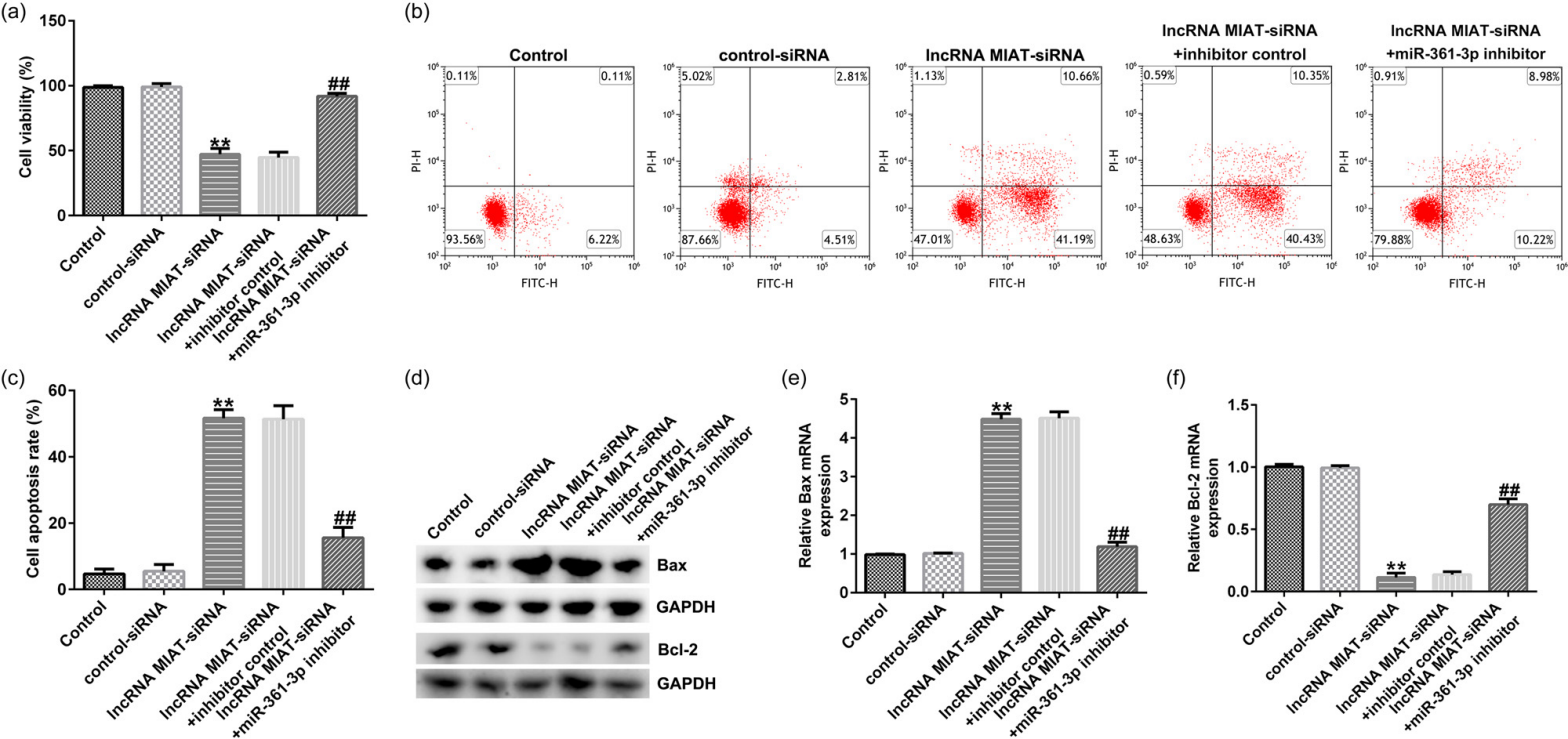
Effects of lncRNA MIAT-siRNA on the growth of Du145 cells. (a) The viability of the transfected Du145 cells was detected by MTT assay. (b and c) The cell apoptosis rates of Du145 cells were detected by FCM analysis. (d) The protein expression levels of Bax and Bcl-2 in Du145 cells were measured by Western blot analysis. (e and f) The mRNA expression levels of Bax and Bcl-2 in Du145 cells were detected by qRT-PCR assay. All experiments were repeated three times, and the data are shown as the mean value ± SD. **p < 0.01 vs control-siRNA; ##*p* < 0.01 vs lncRNA MIAT-siRNA + inhibitor control.

To investigate the effects of lncRNA MIAT in the growth of Du145 cells, the cells were transfected, and then MTT, FCM, Western blot, and qRT-PCR assays were performed. The results showed that, compared with control-siRNA group, lncRNA MIAT-siRNA remarkably inhibited the viability of Du145 cells (Figure 4a) and induced apoptosis (Figure 4b and c). Moreover, as shown in Figure 4d–f, lncRNA MIAT-siRNA significantly improved the protein and mRNA expression of Bax, and reduced the protein and mRNA expression of Bcl-2. All these effects were observably reversed by miR-361-3p inhibitor.

### 3.5 CCAR2 was the target gene of miR-361-3p

**Figure 5:**
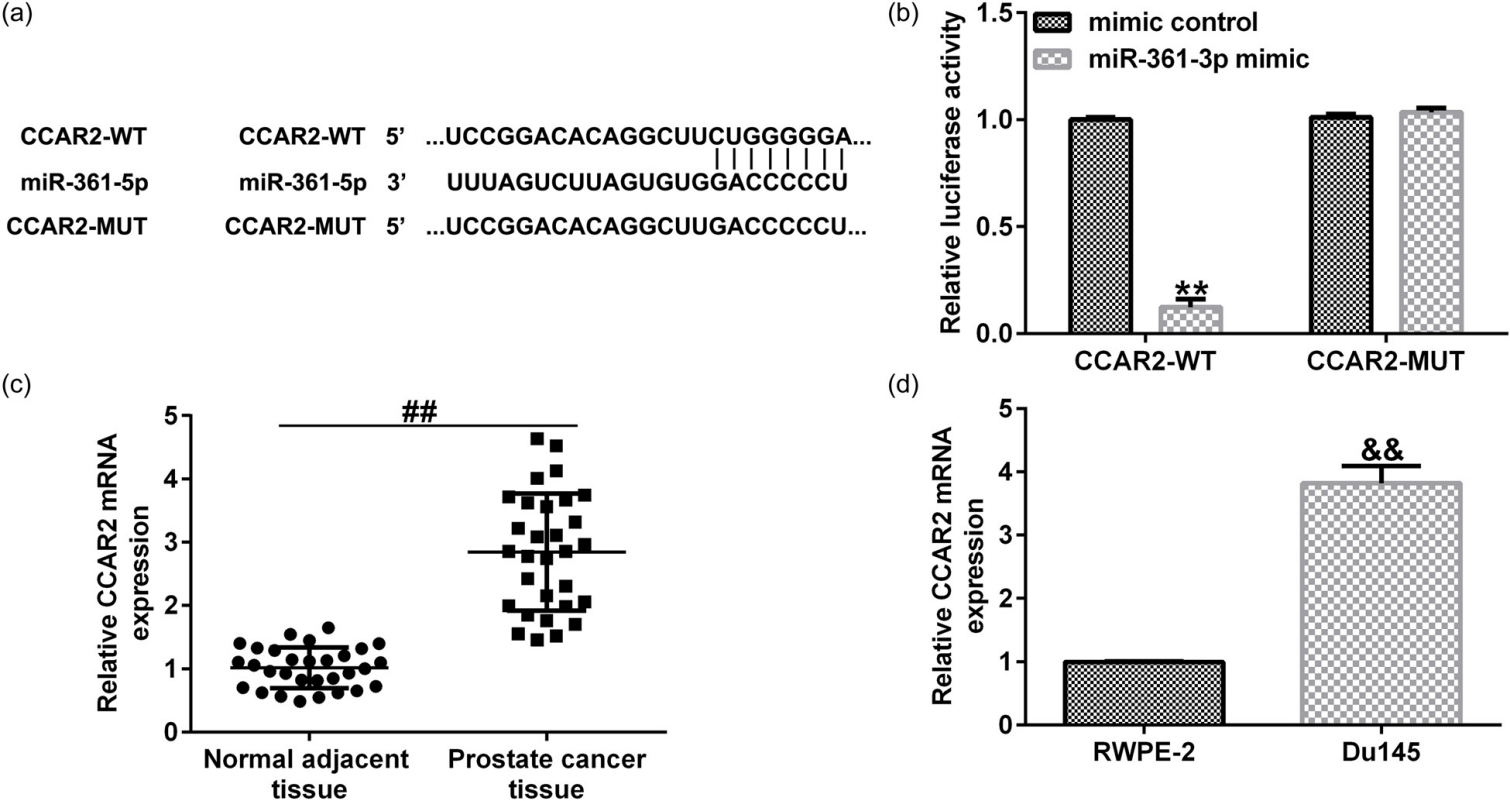
miR-361-3p directly targeted CCAR2. (a) TargetScan predicted the binding site between CCAR2 and miR-361-3p. (b) Dual-luciferase reporter assay verified the binding site between CCAR2 and miR-361-3p. (c and d) qRT-PCR analysis was performed to measure the mRNA expression of CCAR2 in prostate cancer tissues and cells. All experiments were repeated three times, and the data are shown as the mean value ± SD. **p < 0.01 vs mimic control; ##*p* < 0.01 vs Normal adjacent tissue; &&*p* < 0.01 vs RWPE-2.

To further study the relevant mechanism of action, we explored the relationship between CCAR2 and miR-361-3p. The result of TargetScan analysis showed that there was a potential binding site between CCAR2 and miR-361-3p (Figure 5a), and the binding site was confirmed by dual-luciferase reporter assay (Figure 5b). Besides, compared with adjacent tissues, the mRNA expression of CCAR2 in prostate cancer tissues was significantly upregulated (Figure 5c). Similarly, the mRNA expression of CCAR2 in the prostate cancer cell line Du145 cells was significantly increased compared with normal human prostate epithelial cells RWPE-2 cells (Figure 5d).

### 3.6 miR-361-3p negatively regulated CCAR2 expression in Du145 cells

**Figure 6:**
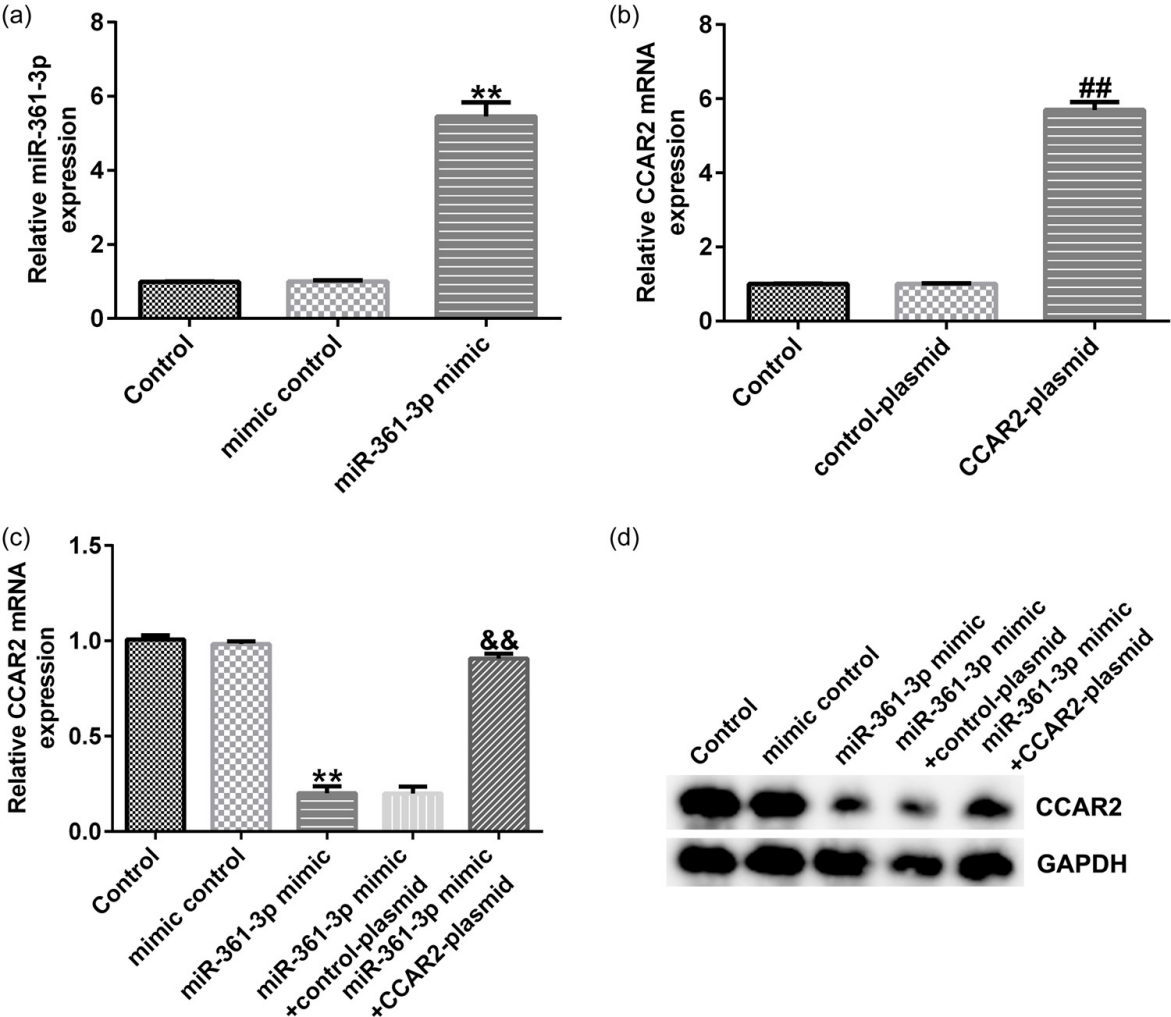
Effects of miR-361-3p on the expression of CCAR2 in Du145 cells. (a–c) qRT-PCR analysis was used to detect the mRNA expression of CCAR2 and miR-361-3p in the transfected Du145cells. (d) Western blot analysis was performed to detect the protein expression of CCAR2 in Du145 cells. All experiments were repeated three times, and the data are shown as the mean value ± SD. **p < 0.01 vs mimic control; ##*p* < 0.01 vs control-plasmid; &&*p < 0.01 vs miR-361-3p mimic + control-plasmid.

Transfection efficiency was detected after the cells were transfected for 48 h. As shown in Figure 6a and b, CCAR2-plasmid and miR-361-3p mimic significantly increased the mRNA expression of CCAR and miR-361-3p, respectively, in Du145 cells. Compared with mimic control group, miR-361-3p mimic significantly decreased the mRNA and protein expression of CCAR2 in Du145 cells, and the effects were reversed by CCAR2-plasmid (Figure 6c and d).

### 3.7 miR-361-3p affected the growth of Du145 cells by regulating CCAR2

**Figure 7:**
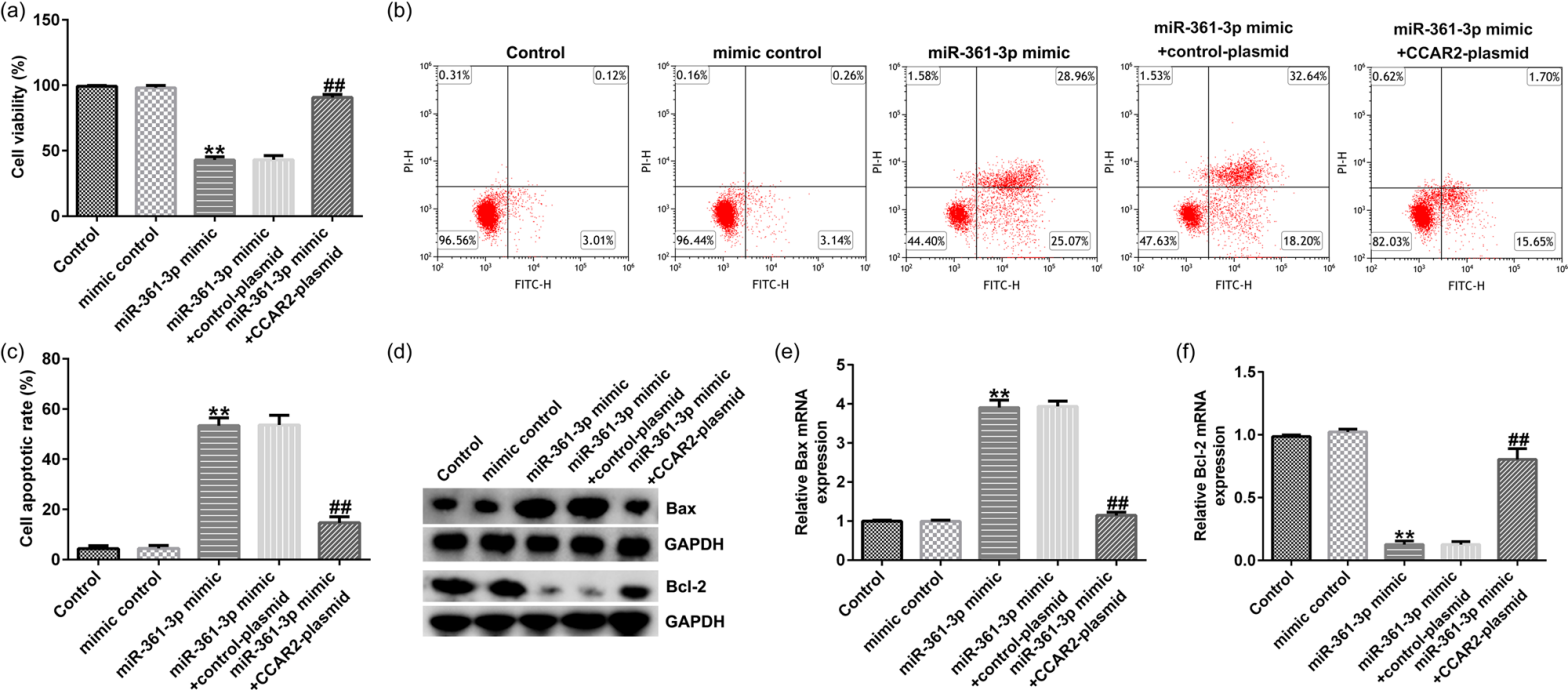
Effects of miR-361-3p mimic on the growth of Du145 cells. (a) The viability of the transfected Du145 cells was detected by MTT assay. (b and c) The cell apoptosis rates of Du145 cells were detected by FCM analysis. (d) The protein expression levels of Bax and Bcl-2 in Du145 cells were measured by Western blot analysis. (e and f) The mRNA expression levels of Bax and Bcl-2 in Du145 cells were detected by qRT-PCR assay. All experiments were repeated three times, and the data are shown as the mean value ± SD. **p < 0.01 vs mimic control; ##*p* < 0.01 vs miR-361-3p mimic + control-plasmid.

The results of MTT and FCM analysis showed that, compared with mimic control group, miR-361-3p mimic significantly decreased the viability (Figure 7a) and induced apoptosis (Figure 7b and c) of Du145 cells. In addition, as shown in Figure 7d–f, miR-361-mimic could increase the protein and mRNA expression of Bax, and reduce the protein and mRNA expression of Bcl-2. All the effects were observably reversed by CCAR2-plasmid.

### 3.8 CCAR2 reversed the effects of lncRNA MIAT-siRNA on Du145 cells

**Figure 8:**
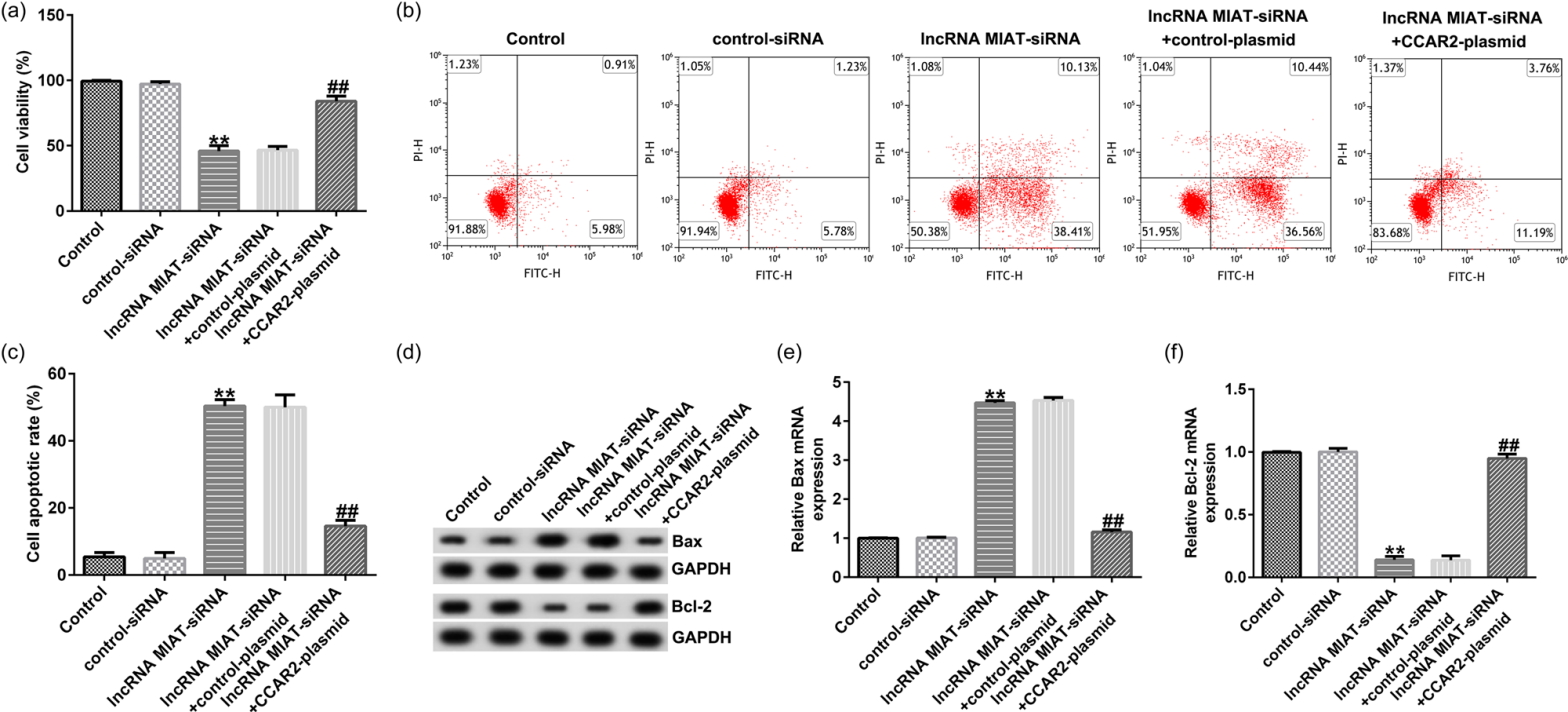
CCAR2 reversed the effects of lncRNA MIAT-siRNA on Du145 cells. Du145 cells were transfected with control-siRNA, lncRNA MIAT-siRNA, lncRNA MIAT-siRNA + control-plasmid, or lncRNA MIAT-siRNA + CCAR2 plasmid for 48 h. (a) The viability of the transfected Du145 cells was detected by MTT assay. (b and c) The cell apoptosis rates of Du145 cells were detected by FCM analysis. (d) The protein expression levels of Bax and Bcl-2 in Du145 cells were measured by Western blot analysis. (e and f) The mRNA expression levels of Bax and Bcl-2 in Du145 cells were detected by qRT-PCR assay. All experiments were repeated three times, and the data are shown as the mean value ± SD. **p < 0.01 vs control-siRNA; ##*p* < 0.01 vs lncRNA MIAT-siRNA + control-plasmid.

Finally, we explored whether CCAR2-plasmid could reverse the effects of lncRNA MIAT-siRNA on Du145 cells. Du145 cells were transfected with control-siRNA, lncRNA MIAT-siRNA, lncRNA MIAT-siRNA + control-plasmid, or lncRNA MIAT-siRNA + CCAR2 plasmid for 48 h. Then, cell viability and cell apoptosis were determined using MTT and FCM analysis. Results revealed that lncRNA MIAT-siRNA induced Du145 cell viability reduction and apoptosis induction were significantly reversed by CCAR2-plasmid (Figure 8a–c). Moreover, Western blot assay and qRT-PCR indicated that lncRNA MIAT-siRNA induced Bax protein and mRNA upregulation and Bcl-2 protein and mRNA reduction were significantly abolished by CCAR2-plasmid (Figure 8d–f). These findings indicated that CCAR2 could reverse the effects of lncRNA MIAT-siRNA on Du145 cells

## 4 Discussion

Prostate cancer is the major cause of death for men worldwide [22]. Therapeutic options are represented by surgery, radiotherapy, hormonotherapy, and chemotherapy stand-alone or in a combination approach. The challenge nowadays is to better understand the mechanism of cancer development [23], finding real prostate cancer markers, and developing new specific therapies in order to reduce the mortality of prostate cancer. In this sense, the research on lncRNA/miRNA is of paramount importance as they could be novel diagnostic/therapeutic instruments as biomarkers for the diagnosis and possible targets for new therapies [24,25,26].

Studies have shown that miR-361-3p was involved in regulating the occurrence and development of prostate cancer, and lncRNA MIAT took part in many cancers [20,27,28]. We thus suspected that lncRNA MIAT was associated with the progress of prostate cancer. In this research, we detected the expression of lncRNA MIAT and miR-361-3p in prostate cancer tissues and adjacent nontumor tissues, and found that lncRNA MIAT was upregulated in the prostate cancer tissues compared with adjacent nontumor tissues, which was opposite to the expression level of miR-361-3p in the prostate cancer tissues. Similarly, lncRNA MIAT expression was increased in prostate cancer cells Du145 and miR-361-3p expression was decreased. However, only one prostate cell line and one normal cell line were used to show the expression difference of lncRNA MIAT and miR-361-3p between normal and prostate cells. This was a limitation of current study.

In order to further study the mechanism of lncRNA MIAT in prostate cancer, we predicted and verified the binding sites between lncRNA MIAT and miR-361-3p. In addition, we found that lncRNA MIAT negatively regulated the expression of miR-361-3p in Du145 cells. Downregulation of lncRNA MIAT inhibited the cell viability and induced the cell apoptosis of prostate cancer cells (Du145 cells) by upregulating the expression of miR-361-3p. B-cell lymphoma/leukemia-2 (Bcl-2) is a proto-oncogene, which has a significant effect on inhibiting cell apoptosis [29]. Bax is the most widely studied pro-apoptotic protein in the Bcl-2 family, and the expression of Bax is elevated during apoptosis in many cells [30]. In the present study, the expression of Bax was improved and Bcl-2 expression was decreased when downregulating lncRNA MIAT, which provided evidence for the pro-apoptotic effect of lncRNA MIAT-siRNA. Similarly, we verified the relationship between miR-361-3p and CCAR2 and confirmed the upregulation of CCAR2 in prostate cancer. To assess whether miR-361-3p affected the cell growth of prostate cancer cells by regulating CCAR2, we performed the experiments in Du145 cells after upregulating miR-361-3p and CCAR2. The results showed that miR-361-3p inhibited cell viability and promoted cell apoptosis of Du145 cells by downregulating CCAR2 expression. It was worth mentioning that CCAR2 reversed all the effects of lncRNA MIAT-siRNA on Du145 cells.

In conclusion, we found that lncRNA MIAT knockdown inhibited cell proliferation and induced apoptosis in prostate cancer through miR-361-3p/CCAR2 axis and participated in prostate cancer, which would provide new targets for the treatment of prostate cancer.
